# Detailed description of *Senegalia massiliensis* strain SIT17^T^*,* a bacterium isolated from the human gut

**DOI:** 10.1016/j.nmni.2020.100700

**Published:** 2020-05-23

**Authors:** S.I. Traore, I.I. Ngom, C.I. Lo, F. Di Pinto, C. Sokhna, P.-E. Fournier, D. Raoult, F. Fenollar

**Affiliations:** 1) Aix Marseille Univ, IRD, AP-HM, MEФI, Marseille, France; 2) IHU-Méditerranée Infection, Marseille, France; 3) Aix Marseille Univ, IRD, AP-HM, SSA, VITROME, Marseille, France; 4) Campus Commun UCAD-IRD of Hann, Dakar, Senegal

**Keywords:** Culturomics, human gut, *Senegalia massiliensis*, taxonogenomics, bacteria

## Abstract

Strain SIT17^T^ was isolated from the stool of a healthy 13-month-old Senegalese boy. It is a Gram-positive, anaerobic, rod-shaped, non-spore-forming and mobile bacterium. It exhibited 92.74% 16S rRNA gene sequence similarity with the *Brassicibacter thermophilus* strain Cel2f, the phylogenetically most closely related species. Its genome is about 2.87 Mb long with 27.39 mol% G + C content. We provide more details of *Senegalia massiliensis* strain SIT17^T^ (= CSURP2130 = DSM 103071), the creation of which was previously announced.

## Introduction

Recently, the culturomics concept developed in our laboratory has allowed us to change the paradigm of the human gut microbiota [1]. Indeed, by this method, >50% of the microorganisms present in the human gut microbiota are known [2]. To improve culture and bacterial identification, culturomics is associated with a new process named taxonogenomics to provide exhaustive information and to better characterize bacterial species [[3], [4], [5]]. Combining phenotypic characteristics and genomic analysis and comparison, this polyphasic approach exceeds the limits of conventional methods long used for the description of new species [[6], [7], [8]].

Here, we present the classification and features of *Senegalia massiliensis* strain SIT17^T^, including a description of the complete genome sequencing and annotation.

### Isolation and growth conditions

**Fig. 1:**
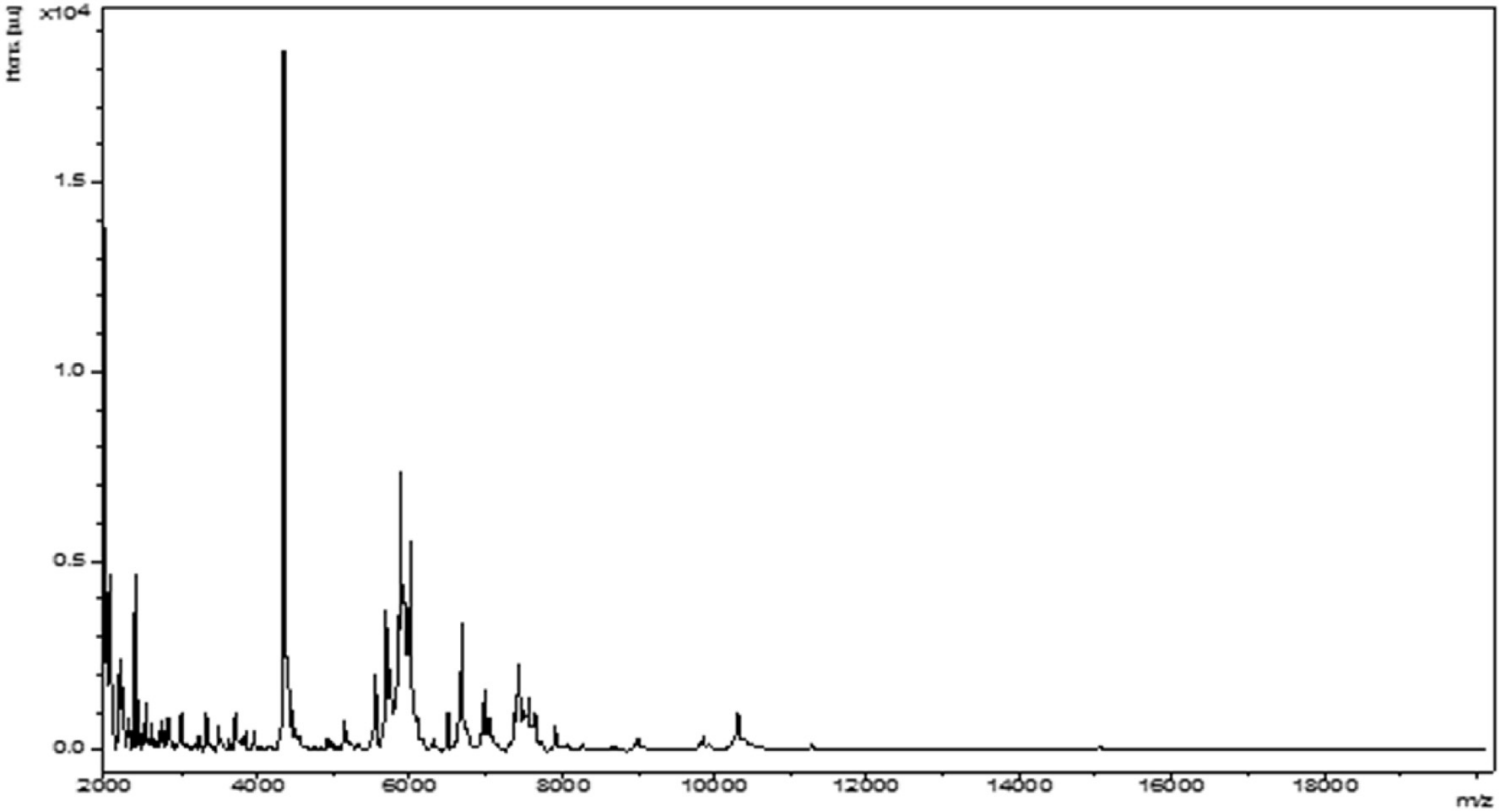
Reference mass spectrum from *Senegalia massiliensis* strain SIT17^T^.

Strain SIT17^T^ was first isolated in 2015 from the stool of a healthy 13-month-old Senegalese boy [9]. The sample was collected in Senegal and was then frozen at –80°C. Subsequently, it was transported in dry ice to Marseille, where the bacterial culture was started. The initial growth of bacterial cells was obtained on Columbia agar with 5% sheep's blood after 2 days of anaerobic incubation at 37°C. The identification of strain SIT17^T^ using matrix assisted laser desorption/ionization time-of-flight mass spectrometry was unsuccessful. The process was performed on a Microflex LT spectrometer (Bruker, Daltonics, Bremen, Germany) as previously described [[10], [11]]. The spectra obtained were imported and analysed using the Biotyper 3.0 software against the Bruker database, which is permanently improved with the local MEPHI database (Fig. 1).

### Strain identification and phylogenetic analysis

**Fig. 2:**
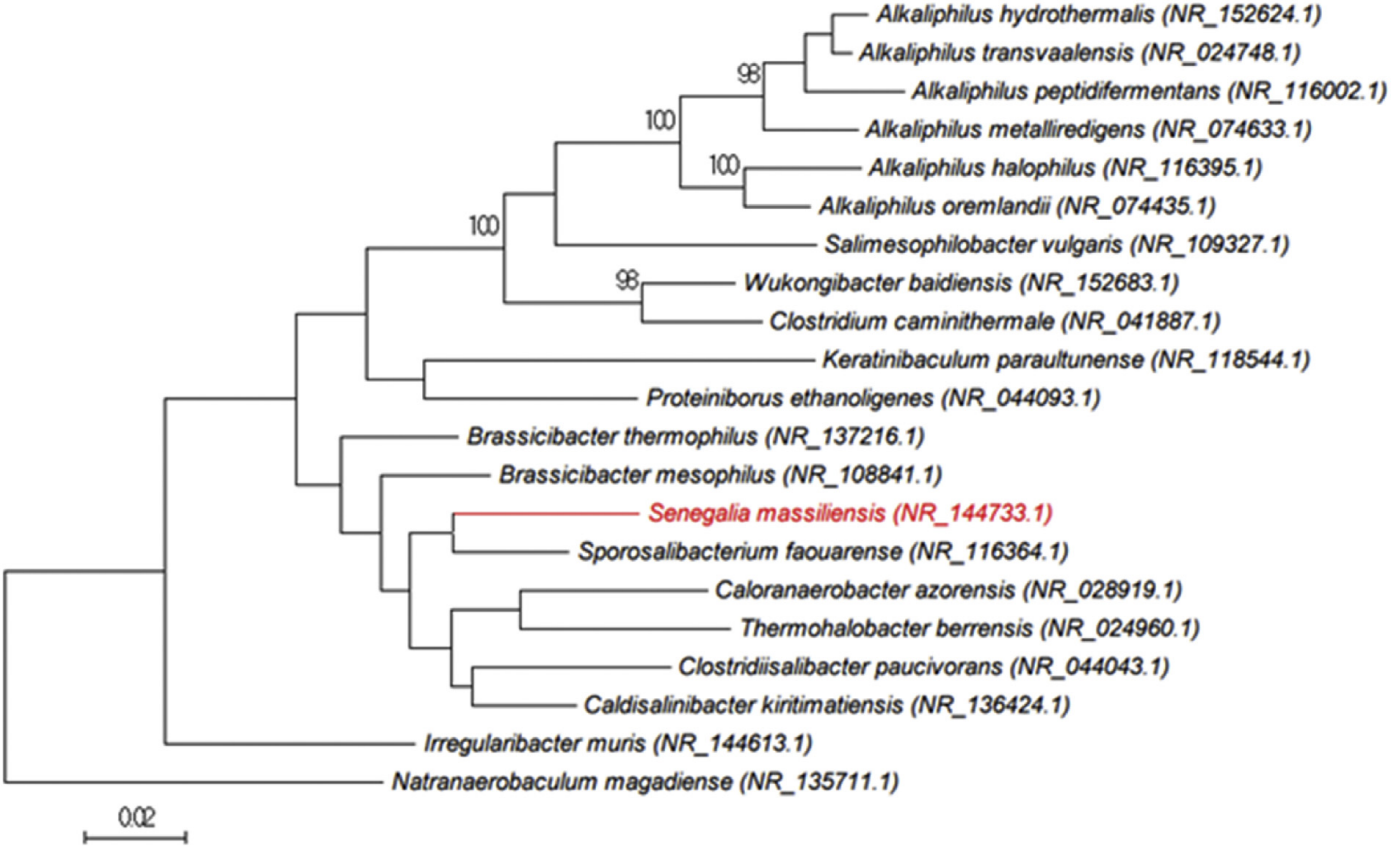
Phylogenetic tree highlighting the position of *Senegalia massiliensis* strain SIT17^T^ relative to other close species. Sequences were aligned using CLUSTALW, with default parameters, and phylogenetic inferences were obtained using the maximum likelihood method within the MEGA7 software. Numbers at the nodes are percentages of bootstrap values obtained by repeating the analysis 500 times to generate a majority consensus tree. The scale bar indicates a 2% nucleotide sequence divergence.

In order to identify the strain SIT17^T^, the 16S rRNA gene was amplified using the fD1 and rP2 primer pair (Eurogentec, Angers, France) and sequenced using the Big Dye® Terminator v1.1 Cycle Sequencing Kit and 3500xLGenetic Analyzer capillary sequencer (Thermofisher, Saint-Aubin, France), as previously reported [12]. The 16S rRNA nucleotide sequences were assembled and corrected using CodonCode Aligner software (http://www.codoncode.com). The PCR-amplified genes coding for 16S rRNA of *Senegalia massiliensis* yielded 92.74% similarity level with *Brassicibacter thermophilus* strain Cel2f (GenBank accession no: NR137216) [13], the phylogenetically closest species with standing in nomenclature (Fig. 2). This value was lower than 95%, which is the recommended threshold for delineating a new bacterial genus based on 16S rRNA gene sequence without DNA–DNA hybridization [[14], [15]]. Classification and general features are summarized in Table 1.

**Table 1:** Classification and general features of *Senegalia massiliensis* strain SIT17^T^

Property	Terms
Current classification	Domain: *Bacteria*
	Phylum: *Firmicutes*
	Class: *Clostridia*
	Order: *Clostridiales*
	Family: *Clostridiaceae*
	Genus: *Senegalia*
	Species: *Senegalia massiliensis*
	Type: strain SIT17^T^
Gram stain	Positive
Cell shape	Rod
Motility	Motile
Sporulation	Not sporulating
Temperature range	28°C–45°C
Optimum temperature	37°C
pH range (optimum)	7
Oxygen requirement	Anaerobic
Carbon source	Unknown
Habitat	Human gut
Biotic relationship	Free-living
Pathogenicity	Unknown

### Phenotypic and biochemical characteristics

**Fig. 3:**
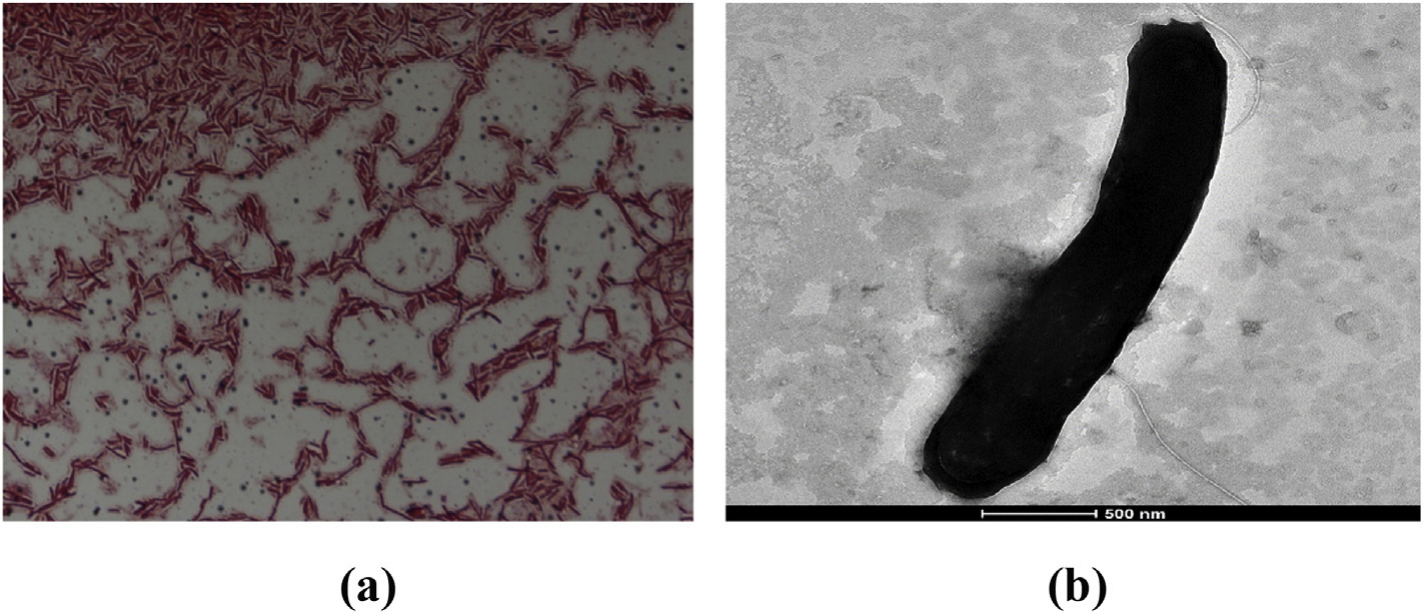
The morphology of bacterial cells of strain SIT17^T^. (a) Gram staining of *Senegalia massiliensis* strain SIT17^T^. (b) Transmission electron microscopy of *Senegalia massiliensis* strain SIT17^T^ using Tecnai G20 electron microscope (FEI Company). The scale bar represents 500 nm.

Colonies of the strain SIT17^T^ were grey and translucent with a size of 0.5–1 mm on Columbia agar with 5% sheep's blood. Growth was observed from 28°C to 45°C, with optimal growth at 37°C, and colonies were obtained after 48 hours of culture. Bacterial cells were Gram-positive, rod-shaped and motile, but non-spore-forming (Fig. 3a). Observed under electronic microscopy, the cells presented a mean diameter of 0.4 μm and a mean length of 3.2 μm (Fig. 3b). The bacterium was catalase positive but had no oxidase activity. *Senegalia massiliensis* is able to grow in an environment with a pH ranging from 6 to 8.5, with an optimal value of 7. Strain SIT17^T^ is an anaerobic bacterium that can grow in a microaerophilic atmosphere. On the other hand, no growth was observed under aerobic conditions. The biochemical and phenotypic features of strain SIT17^T^ were compared with those of other close representative strains in the *Clostridiaceae* family (Table 2). Using API ZYM strips (bioMérieux, Marcy l’Étoile, France), positive reactions were observed for esterase, esterase lipase, alkaline phosphatase, α-chymotrypsin, acid phosphatase, naphthol-AS-BI-phosphohydrolase and β-galactosidase. However, we noted that the enzymatic activities for lipase, leucine arylamidase, valine arylamidase, cystine arylamidase, trypsin, α-galactosidase, β-glucuronidase, β-glucosidase, *N*-acetyl-β-glucosaminidase, α-mannosidase and α-fucosidase, were negative. Using API 50 CH, positive reactions were observed for glycerol, d-ribose, l-xylose, d-galactose, d-glucose, d-fructose, d-mannose, l-rhamnose, inositol, d-mannitol, d-sorbitol, methyl α-d-glucopyranoside, *N*-acetylglucosamine, amygdalin, arbutin, salicin, d-cellobiose, d-maltose, d-lactose, d-sucrose, d-trehalose, d-melezitose, d-raffinose, d-turanose, d-xylose, d-fucose, l-fucose, d-arabitol, potassium gluconate and starch. However, there was no metabolism for the following carbohydrates: erythritol, l-arabinose, d-adonitol, methyl β-d-xylopyranoside, methyl α-d-mannopyranoside, d-arabinose, inulin and glycogen. Cellular fatty acid methyl esters analysis of the strain SIT17^T^ was carried out by operating gas chromatography/mass spectrometry as previously described [[16], [17]]. The result showed that hexadecanoic acid (32.6%), 9-octadecenoic acid (21.6%) and 13-methyl-tetradecanoic acid (11.9%), are the most abundant fatty acids. Other saturated and unsaturated fatty acids are also found (Table 3).

**Table 2:** Differential characteristics of *Senegalia massiliensis* strain SIT17^T^ (data from this study) compared to other close bacteria

Properties	*Senegalia massiliensis*	*Clostridiisalibacter paucivorans*	*Alkaliphilus oremlandii*	*Alkaliphilus transvaalensis*	*Proteiniborus ethanoligenes*	*Sporosalibacterium faouarense*
Cell diameter (μm)	0.3–0.5	0.5	0.5	0.4–0.7	0.5–0.6	0.5
Oxygen requirement	Anaerobic	Anaerobic	Anaerobic	Anaerobic	Anaerobic	Anaerobic
Shape	bacilli	bacilli	bacilli	bacilli	bacilli	bacilli
Gram stain	+	+	+	+	+	+
Motility	+	—	+	+	—	+
Indole	—	—	NA	+	—	NA
Production of:						
Alkaline phosphatase	+	NA	NA	NA	NA	NA
Catalase	+	NA	NA	NA	NA	NA
Oxidase	—	NA	NA	NA	NA	NA
Nitrate reductase	—	NA	—	+	+	NA
Urease	—	NA	NA	NA	NA	NA
β-galactosidase	+	NA	NA	NA	NA	NA
*N*-acetyl glucosamine	—	NA	NA	NA	NA	NA
Acid from:						
l-arabinose	—	+	NA	NA	—	+
Ribose	+	—	NA	—	—	—
Mannose	+	—	NA	—	—	—
Mannitol	+	NA	NA	NA	—	+
d-glucose	—	NA	NA	—	—	+
d-fructose	+	—	+	—	—	+
d-maltose	+	NA	NA	—	—	—
d-lactose	+	NA	NA	—	—	—
G + C content (%)	27.4	33.0	36.1	36.4	38.0	37.7
Habitat	Human colon	Wastewater	Environment	Environment	Environment	Soil

−, negative reaction; +, positive reaction; NA, not available data.

**Table 3:** Cellular fatty acid profiles (%) of *Senegalia massiliensis* strain SIT17^T^ compared with other species

Fatty acids	Names	1	2	3	4	5
12:00	Dodecanoic acid	1.2	—	—	—	—
13:00	Tridecanoic acid	TR	—	2.3	—	4.4
14:00	Tetradecanoic acid	9.2	14.3	1.7	15.58	21.6
15:0 anteiso	12-methyl-tetradecanoic acid	1.0	1.5	2.8	—	3.9
15:0 iso	13-methyl-tetradecanoic acid	11.9	6.6	51.6	4.30	41
16:00	Hexadecanoic acid	32.6	7.6	3.9	25.40	1.2
16:1n5	11-Hexadecanoic acid	TR	—	1.9	6.18	—
17:00	Heptadecanoic acid	TR	—	—	—	0.6
17:1n7	10-Heptadecenoic acid	TR	19.3	12.2	9.49	—
18:00	Octadecanoic acid	4.7	—	7.2	12.03	1.3
18:1n7	11-Octadecenoic acid	1.7	—	2.0	—	—
18:1n9	9-Octadecenoic acid	21.6	—	1.1	11.20	—

1, *Senegalia massiliensis* strain SIT17^T^; 2, *Clostridiisalibacter paucivorans* strain 37HS60^T^ [19]; 3, *Alkaliphilus transvaalensis* strain SAGM1^T^ [20]; 4, *Proteiniborus ethanoligenes* strain GW^T^ [21]; 5, *Sporosalibacterium faouarense* strain SOL3f37^T^ [22].

TR, trace amounts <1%; –, not detected.

### Genomic properties and comparison

**Fig. 4:**
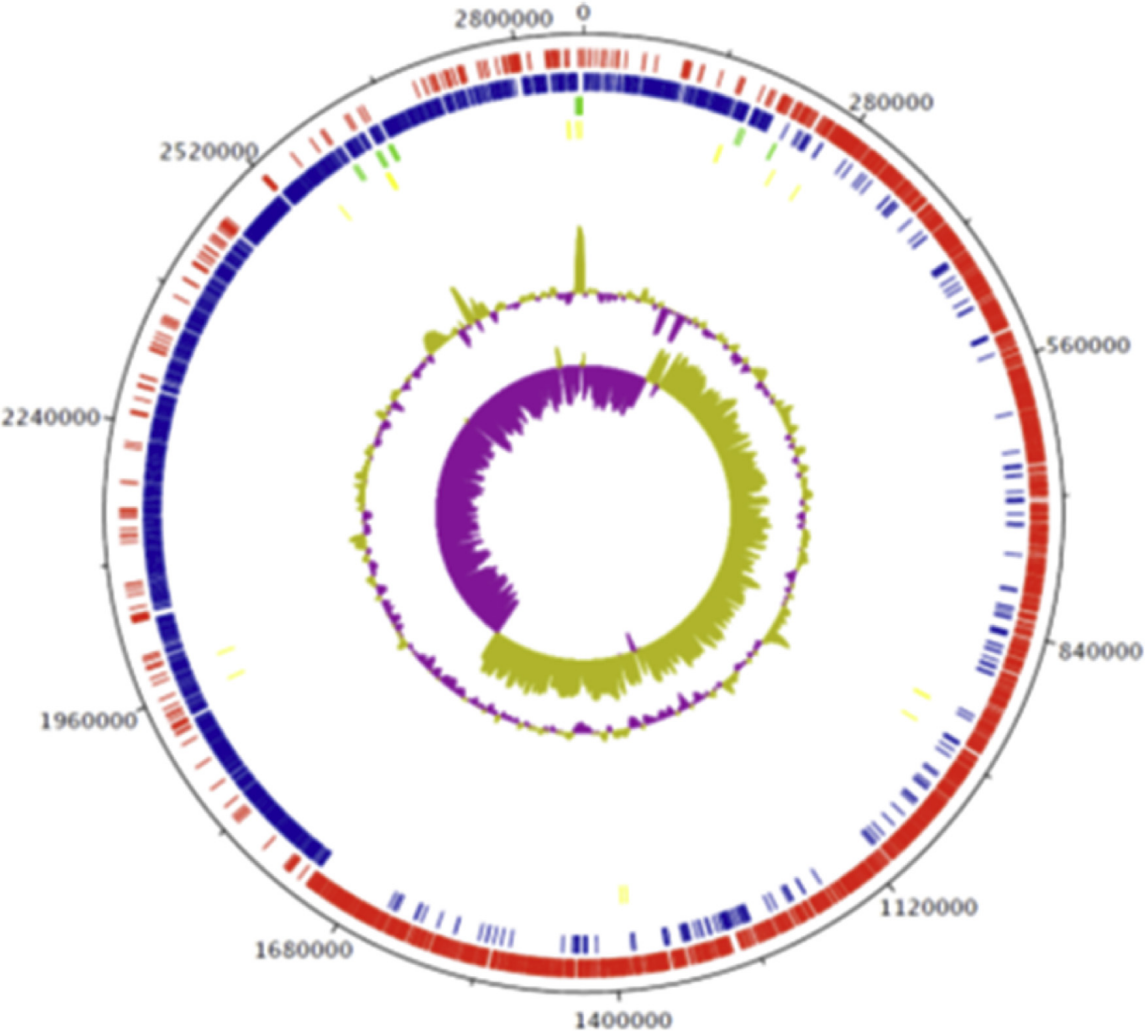
Graphical circular map of the chromosome. From outside to the centre: Contigs (red), cluster of orthologous groups (COG) category of genes on the forward strand (three circles), genes on forward strand (blue circle), genes on the reverse strand (red circle), COG category on the reverse strand (three circles), G + C content.

**Fig. 5:**
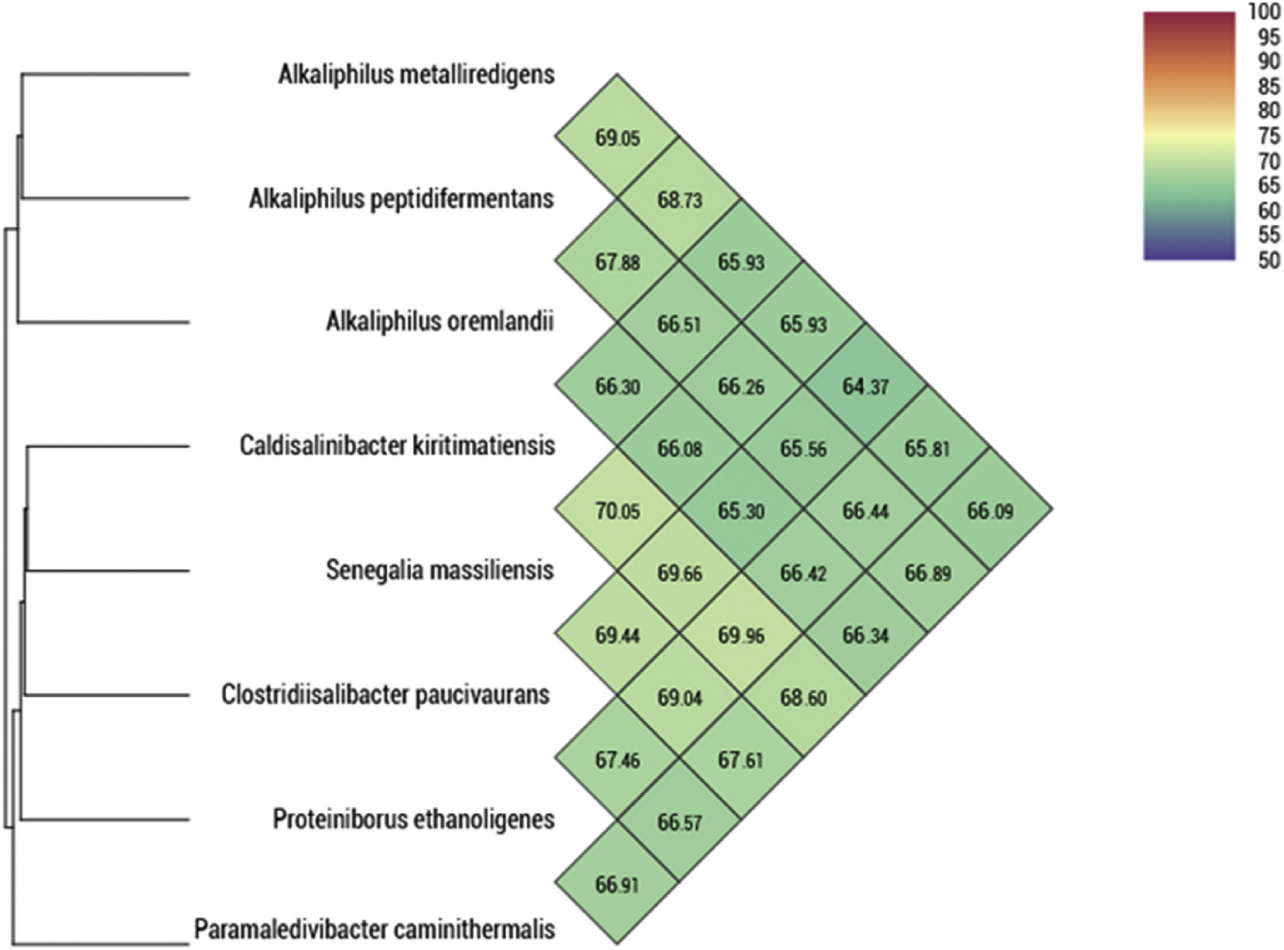
Heatmap generated with OrthoANI values calculated using the OAT software between *Senegalia massiliensis* and other closely related species with standing in nomenclature.

The genome of strain SIT17^T^ is 2 866 883 bp long with 27.39 mol% G + C content and it contains 2933 coding genes (Fig. 4). It is composed of 22 contigs. By comparing it with related species, its genome (2.87 Mbp) is smaller than those of *Alkaliphilus oremlandii* strain OhILAsm, *Proteiniborus ethanoligenes* strain DSM 21650, *Clostridiisalibacter paucivorans* strain DSM 22131, *Alkaliphilus transvaalensis* strain ATCC 700919, *Paramaledivibacter caminithermalis* strain DSM 15212, *Alkaliphilus peptidifermentans* strain DSM 18978 and *Alkaliphilus metalliredigens* strain L21-TH-D2 (3.12, 3.16, 3.24, 4.02, 4.05, 4.45 and 4.93 Mbp, respectively), but larger than the genome of *Caldisalinibacter kiritimatiensis* (2.79 Mbp). The G + C content of strain SIT17^T^ (27.39 mol%) is smaller than those of *A. oremlandii*, *Proteiniborus ethanoligenes*, *Clostridiisalibacter paucivorans*, *A. transvaalensis*, *Paramaledivibacter caminithermalis*, *A. peptidifermentans*, *A. metalliredigens* and *Caldisalibacter kiritimatiensis* (36.3, 32.6, 31.4, 34.0, 30.5, 34.1, 36.8 and 30.1 mol%, respectively). The gene content of strain SIT17^T^ (2933 genes) is larger than those of *A. oremlandii* (*n* = 2898), *Caldisalibacter kiritimatiensis* (*n* = 2557) and *Proteiniborus ethanoligenes* (*n* = 2846), but smaller than *Clostridiisalibacter paucivorans* (*n* = 3014), *A. peptidifermentans* (*n* = 4072), *A. metalliredigens* (*n* = 4641), *A. transvaalensis* (*n* = 3640) and *Paramaledivibacter caminithermalis* (*n* = 3543). Distribution of functional classes of predicted genes according to the clusters of orthologous groups is reported in Table 4. Results from pairwise genome comparison obtained from analysis of the digital DNA–DNA hybridization using GGDC software [18] are shown in Table 5. OrthoANI values among the closely related species ranged from 64.37%, between *A. metalliredigens* and *Clostridiisalibacter paucivorans*, to 70.05 % between *Caldisalinibacter kiritimatiensis* and *S. massiliensis*. When *S. massiliensis* was compared with these closely related species, values ranged from 65.93% with *A. metalliredigens* to 70.05% with *Caldisalinibacter kiritimatiensis* (Fig. 5).

**Table 4:** Distribution of functional classes of predicted genes according to the clusters of orthologous groups of proteins of *Senegalia massiliensis* strain SIT17^T^

Code	Value	Description
[J]	244	Translation, ribosomal structure and biogenesis
[A]	0	RNA processing and modification
[K]	225	Transcription
[L]	114	Replication, recombination and repair
[B]	1	Chromatin structure and dynamics
[D]	52	Cell-cycle control, cell division, chromosome partitioning
[Y]	0	Nuclear structure
[V]	80	Defence mechanisms
[T]	181	Signal transduction mechanisms
[M]	148	Cell wall/membrane/envelope biogenesis
[N]	71	Cell motility
[Z]	0	Cytoskeleton
[W]	11	Extracellular structures
[U]	28	Intracellular trafficking, secretion and vesicular transport
[O]	116	Post-translational modification, protein turnover, chaperones
[X]	26	Mobilome: prophages, transposons
[C]	173	Energy production and conversion
[G]	144	Carbohydrate transport and metabolism
[E]	202	Amino acid transport and metabolism
[F]	92	Nucleotide transport and metabolism
[H]	124	Coenzyme transport and metabolism
[I]	89	Lipid transport and metabolism
[P]	145	Inorganic ion transport and metabolism
[Q]	34	Secondary metabolites biosynthesis, transport and catabolism
[R]	255	General function prediction only
[S]	203	Function unknown
─	563	Hypothetical protein

**Table 5:** Pairwise comparison of *Senegalia massiliensis* strain SIT17^T^ with other species using GGDC formula^a^

	*Senegalia massiliensis*	*Alkaliphilus metalliredigens*	*Alkaliphilus oremlandii*	*Alkaliphilus transvaalensis*	*Alkaliphilus peptidifermentans*	*Proteiniborus ethanoligenes*	*Clostridiisalibacter paucivorans*	*Paramaledivibacter caminithermalis*	*Caldisalinibacter kiritimatiensis*
*Caldisalinibacter kiritimatiensis*	19.7 ± 2.4	37.2 ± 5	34 ± 4.9	17.5 ± 4.4	18 ± 4.5	18.9 ± 4.6	16.9 ± 4.4	23.6 ± 4.7	100%
*Paramaledivibacter caminithermalis*	15.2 ± 4.3	28.4 ± 4.9	26.4 ± 4.8	16 ± 4.3	19.5 ± 4.6	19 ± 4.5	23.6 ± 4.7	100%	
*Clostridiisalibacter paucivorans*	17.5 ± 4.5	25 ± 4.8	16.7 ± 4.4	31 ± 4.9	17.8 ± 5.4	18.6 ± 5.5	100%		
*Proteiniborus ethanoligenes*	17.3 ± 4.1	19.9 ± 4.6	19.8 ± 4.6	18.7 ± 4.6	16.2 ± 4.3	100%			
*Alkaliphilus peptidifermentans*	17.2 ± 4.4	25.5 ± 5.2	24.8 ± 4.8	19.9 ± 4.7	100%				
*Alkaliphilus transvaalensis*	27.4 ± 4.8	23.5 ± 4.8	22.2 ± 4.7	100%					
*Alkaliphilus oremlandii*	29.9 ± 4.9	26.8 ± 4.9	100%						
*Alkaliphilus metalliredigens*	33.9 ± 4.9	100%							
*Senegalia massiliensis*	100%								

a GGDC formula 2: (Identities/high-scoring segment pairs (HSPs)). The confidence intervals indicate the inherent uncertainty in estimating DNA–DNA hybridization values from intergenomic distances based on models derived from empirical test data sets (which are always limited in size). These results are in accordance with the 16S rRNA and phylogenomic analyses as well as the GGDC results.

## Conclusion

On the basis of phenotypic, phylogenetic and genomic analyses, we formally propose the creation of *Senegalia massiliensis* gen. nov. sp. nov., that contains the strain SIT17^T^. Hence, the combination of culturomics and taxonogenomics has contributed to a better knowledge of the associated human microorganisms and to better understanding of physiological functioning in health and disease.

### Description of *Senegalia* gen. nov

*Senegalia* (Se.ne.ga.lia. L. gen. n. Senegalia, the Latin name of Senegal, where the stool specimen was collected). Cells are Gram-positive, non spore-forming, motile and anaerobic bacilli. The type species is *Senegalia massiliensis* sp. nov.

### Description of *Senegalia massiliensis* sp. nov

*Senegalia massiliensis* gen. nov., sp. nov. (mas.si.li.en'sis. L. fem. adj., from *massiliensis*, of Massilia, from the Latin name of Marseille where the strain was first isolated). It is classified as a member of the family *Clostridiaceae* within the phylum *Firmicutes*. The strain SIT17^T^ designed the type strain of *Senegalia massiliensis* gen. nov., sp. nov., and was deposited in CSUR (CSURP2130) and DSMZ (DSM 103071) collections. It is a Gram-positive bacillus, motile, catalase-positive, oxidase-negative and non-spore-forming. Strain SIT17^T^ was first isolated from the stool of a healthy 13-month-old Senegalese boy. Its genome is 2 866 883 bp long with 27.39 mol% G + C content and possesses 2933 coding genes. The genome and 16S rRNA sequences of the strain SIT17^T^ are both deposited in GenBank under accession numbers UZAQ00000000 and LN881608, respectively.

## Conflict of interest

The authors declare no conflict of interest.

## Funding sources

This study was supported by the Institut Hospitalo-Universitaire (IHU) Méditerranée Infection, the National Research Agency under the programme *Investissements d'avenir*, reference ANR-10-IAHU-03, the Région Provence-Alpes-Côte d’Azur and European funding FEDER PRIMI.

## Ethics and consent

The child's parents provided signed informed consent and the study was approved by the ethics committee of the Institut Fédératif de Recherche IFR48 under number 09-022.
